# A Comparative Study of the Short Term Cold Resistance Response in Distantly Related *Drosophila* Species: The Role of *regucalcin* and *Frost*

**DOI:** 10.1371/journal.pone.0025520

**Published:** 2011-10-03

**Authors:** Micael Reis, Cristina P. Vieira, Ramiro Morales-Hojas, Bruno Aguiar, Hélder Rocha, Christian Schlötterer, Jorge Vieira

**Affiliations:** 1 IBMC-Instituto de Biologia Celular e Molecular, University of Porto, Porto, Portugal; 2 Institut für Populationsgenetik, Vetmeduni Vienna, Vienna, Austria; Barnard College, Columbia University, United States of America

## Abstract

The molecular basis of short term cold resistance (indexed as chill-coma recovery time) has been mostly addressed in *D. melanogaster*, where candidate genes (*Dca* (also known as *smp-30)* and *Frost* (*Fst*)) have been identified. Nevertheless, in *Drosophila,* the ability to tolerate short term exposure to low temperatures evolved several times independently. Therefore, it is unclear whether variation in the same candidate genes is also responsible for short term cold resistance in distantly related *Drosophila* species. It should be noted that *Dca* is a candidate gene for cold resistance in the *Sophophora* subgenus only, since there is no orthologous gene copy in the *Drosophila* subgenus. Here we show that, in *D. americana* (*Drosophila* subgenus), there is a north-south gradient for a variant at the 5′ non-coding region of *regucalcin* (a *Dca*-like gene; in *D. melanogaster* the proteins encoded by the two genes share 71.9% amino acid identities) but in our *D. americana* F2 association experiment there is no association between this polymorphism and chill-coma recovery times. Moreover, we found no convincing evidence that this gene is up-regulated after cold shock in both *D. americana* and *D. melanogaster*. Size variation in the Fst PEST domain (putatively involved in rapid protein degradation) is observed when comparing distantly related *Drosophila* species, and is associated with short term cold resistance differences in *D. americana*. Nevertheless, this effect is likely through body size variation. Moreover, we show that, even at two hours after cold shock, when up-regulation of this gene is maximal in *D. melanogaster* (about 48 fold expression change), in *D. americana* this gene is only moderately up-regulated (about 3 fold expression change). Our work thus shows that there are important differences regarding the molecular basis of cold resistance in distantly related *Drosophila* species.

## Introduction

How species adapt to daily and seasonal temperature changes is determined by the genetic variation present in local populations [1]. The molecular basis of short term cold resistance has been mostly addressed in *D. melanogaster*, where candidate genes have been identified (see [2], [3] for reviews of candidate genes for cold-resistance in *D. melanogaster*). In this species, short term cold resistance is very often indexed as chill-coma recovery time (the time it takes an individual to recover from chill-coma once it has been returned to non-stressful temperature [3], , although it is unlikely that this index captures all the complexities of the cold response. It should be noted that there is no effect of acclimation on chill-coma recovery times [9].

The ability to tolerate short term exposure to low temperatures seems to have evolved several times in *Drosophila* [4], but it is unclear whether the same genes have been used in the independent evolutions. Moreover, the model species *D. melanogaster,* where most studies regarding this issue have been performed, is originally a tropical species, and it is conceivable that the genetic basis of cold resistance is different in tropical and temperate species. Comparative studies using divergent species are lacking [2], but are needed in order to establish whether the findings reported for the *D. melanogaster* cold response can be extrapolated to other species. In this work we address the role of *Dca-*like genes and *Frost* (*Fst*) [3] in the cold response of species of the subgenus *Drosophila*.

The *Dca* (in *Drosophila* this gene is also known as *senescence marker protein-30; smp-30*) gene has been found to be up-regulated when *D. melanogaster* flies are exposed one day at 15°C [10]. Moreover, in this species, non-coding polymorphisms have been shown to be significantly associated with cold tolerance [11]. This gene has a predicted Ca^(2+)^-binding function and thus may be involved in the regulation of cytosolic Ca^(2+)^ concentration [12]. It should be noted that in mammals the names *regucalcin* and *smp-30* are synonyms (see for instance, the Human alternative gene names for *regucalcin* at ENSEMBL; http://www.ensembl.org). Nevertheless, in *D. melanogaster, regucalcin* and *smp-30* are the names of two distinct genes on Muller's elements A and E, respectively (http://flybase.org) that code for proteins with 71.9% amino acid identities. In this work, we use the nomenclature for *D. melanogaster*.

Recently, it has been shown that *Dca* arose by a duplication event from the ancestral *regucalcin*-like gene after the split of the *Sophophora* and *Drosophila* subgenera but before the *Sophophora* radiation [13]. There is thus, no *Dca* orthologous gene in species of the *Drosophila* subgenus [13]. Nevertheless, given the degree of similarity between *Dca* (*smp-30*) and *regucalcin*, we decided to address the possible involvement of the latter gene in the cold response of species of the *Drosophila* subgenus. It should be noted, however, that after the duplication event, there is an acceleration of the rate of fixation of nonsynonymous substitutions in the branch leading to *Dca*, which could be indicative of the acquisition of a new function [13]. In *D. melanogaster*, *Dca* promoter polymorphisms show latitudinal clines, and are also associated with wing size but not thorax size [14], [15].

In *D. melanogaster*, the *Fst* gene has been shown to be greatly overexpressed after cold shock [10], [16] and Colinet *et al.* [16] have shown that, in this species, this gene is essential for cold tolerance. It should be noted that, unlike heat-shock genes [17], *Fst* expression was not altered after heat stress [18]. Moreover, this gene is included in a QTL for thermo tolerance [5], [19]. These observations clearly show an important involvement of this gene in the cold response in *D. melanogaster*. Despite its important role in the *D. melanogaster* cold response, length variation in the promoter region of this gene seems to explain at most 1% of the variation in cold-resistance [3]. The *D. melanogaster* Fst protein resembles a mucin-like protein. Like typical mucins it contains multiple tandem repeats rich in serine (S), threonine (T) and proline (P) [16], located in the C terminus of the protein, where Goto [10] identified nine PEEST motives (not identified by this author as PEST (putatively involved in rapid protein degradation) domains). Indeed, Goto [10] identified moderate and weak PEST domains at the N-terminal region of Fst only. Moreover, like secreted mucins, Fst shows an 18 amino acid signal peptide in the N-terminal region [10]. This protein may thus be directed into the endoplasmatic reticulum and secreted into extracellular space [10]. Homology searches revealed similarity with two *D. melanogaster* mucins: *Mur18B* and *Muc11A* [16] that, like *Fst*, show a mRNA enrichment in adult malpighian tubule [20], [21]. Fst may help keep membrane integrity by protecting it from oxidative stress and by allowing the reestablishment of local molecular environments with respect to hydration, ionic composition and concentration. Such injuries are typical features of chilling-injury [16]. In addition to cold tolerance, *Fst* has been reported to respond weakly to abiotic stressors [18], [22], [23], [24], [25] and to be involved in the immune response against virus, bacteria and fungi [26], [27], [28], [29].

Building on the previous work in *D. melanogaster*, we determine the possible involvement of variation in *Fst* and *regucalcin* in the short term cold resistance response in *D. americana*, a temperate species of the *virilis* group of *Drosophila* that has been diverging from *D. melanogaster* for about 40 My [30]. *D. americana* inhabits the Central and Eastern regions from the South (Texas to the states around the Gulf of Mexico) to the North of the country (from Montana to Maine) [31]. The wide geographic distribution of *D. americana* means that individuals from this species face very different climatic conditions.

## Materials and Methods

### Strains

The following 64 *D. americana* strains were used: NN97.2, NN97.4, NN97.9 (Nebraska, USA); G96.11, G96.21, G96.36, G96.48 (Indiana, USA); LP97.7, ML97.4 .2, ML97.5 (Louisiana, USA) and CD97.5 (Louisiana, USA). These strains were originally collected by Bryant McAllister (Iowa University, USA) in the years 1996 and 1997. Furthermore, isofemale strains established with flies collected at the end of July and beginning of August 2004 from four collection sites in the USA, Howell Island, Missouri (HI1, HI13, HI14, HI15, HI18, HI23, HI25, HI27, HI29), Lake Wappapelo, Missouri (W4, W10, W11, W18, W23,W25, W26, W27, W28, W29, W33, W36, W37, W42, W46), Lake Ashbaugh, Arkansas (LA10, LA14, LA18, LA37); Lake Hurricane, Mississippi (H5) and isofemale strains established with flies collected at the end of July 2008 at Fremont (Nebraska) (O27, O28, O29, O30, O31, O32, O33, O34, O35, O37, O38, O39, O40, 042, O45, O47, O50, O53, O57, O61, O62, O64, O66, O69) were also used. The *Drosophila melanogaster* strain Oregon-R was also used in the gene expression experiment described below.

### PCR amplification, cloning and DNA sequencing

Genomic DNA from single males was extracted using the QIAamp DNA Mini Kit from QIAGEN (Izasa Portugal, Lda.). The coding region of *regucalcin* was amplified using primers Dcaf and Dcar (Table S1) designed based on the *D. virilis regucalcin* 5′ flanking and coding regions. Standard amplification conditions were 35 cycles of denaturation at +94°C for 30s, primer annealing at +53°C for 45s, and primer extension at +72°C for 3 min. A *Dca* paralogous gene (*regucalcin*) is here studied, since there is no *Dca* orthologous gene in species of the subgenus *Drosophila* (see Introduction).

An amplification product was obtained for 18 *D. americana* individuals (data not shown). The PCR products were cloned using the TOPO-TA Cloning Kit for Sequencing (Invitrogen, Spain). Positive colonies were picked randomly, grown in 5 mL of LB with Ampicillin, and plasmids were extracted using the QIAprep Spin Miniprep Kit from QIAGEN (Izasa, Portugal). Four colonies were sequenced in order to correct for possible nucleotide missincorporations that may have occurred during the PCR reaction. Sequencing was performed using ABI PRISM Big Dye cycle-sequencing kit version 1.1 (Perkin Elmer, CA, USA) and the primers for the M13 forward and reverse priming sites of the pCR2.1 vector. Sequencing runs were performed by STABVIDA (Portugal). A common polymorphism was observed at the *regucalcin* site −58 which was then used as a molecular marker (see below).

The *Fst* coding region was amplified using primers FrostF and FrostR designed based on the *D. virilis Fst* 5′ and 3′ flanking sequence (Table S1). Standard amplification conditions were those described above. In order to have sequence data for the full spectra of *D. americana Fst* allele sizes found in the 64 *D. americana* strains analyzed (see results), the complete coding sequence of at least one allele of the autosomal *Fst* gene was determined in the following strains, using an approach similar to that described for *regucalcin* (the relative *Fst* allele size, due to different PEST region sizes, is indicated within brackets): NN97.4 (M1), W4 (M1), W11 (M1), NN97.9 (M2), ML97.3 (M2), W29 (M2), ML97.5 (M2), NN97.2 (M3), NN97.8 (M3), LA18 (M3), HI25 (M3), H5 (M3 and M3.5), LP97.7 (M4), HI1 (M4), W11 (M4), ML97.4.2 (M4), LA37 (M5), HI23 (M5), and W37 (M6). DNA sequences were deposited in GenBank (accession numbers JN674303-JN674322).

### regucalcin and Fst genotyping

Because of the possibility of lab adaptation, it is always best to estimate polymorphism frequencies using a sample of wild caught individuals. Therefore, in order to determine the frequency and distribution of the *regucalcin* polymorphism at site −58 in natural populations, 56 wild-caught males and 26 male progeny of inseminated wild-caught females from the *D. americana* populations described in [32], plus 18 wild-caught male individuals from Fremont (Nebraska, July, 2008), 9 wild-caught male individuals from Saint Francisville (Louisiana, July, 2010) and the 4 males, progeny of inseminated wild-caught females from the same population, were used. These individuals were screened for the *regucalcin* −58 polymorphism using the amplification product obtained with Dcaf and Dcar and restriction enzymes *Ava*II plus *Bst*BI. DNA fragments were run in a 2% agarose gel stained with ethidium bromide, using SGTB (Grisp, Portugal) buffer. It should be noted that the *regucalcin* −58 polymorphism is distinguished solely with *BstB*I. Nevertheless it is difficult to distinguish small size differences when DNA fragments are large, as it is here the case (the polymorphism is located at about 40 bp from the 5'end of the amplification product). Therefore, it was necessary to use *Ava*II, which recognizes a shared variant, in order to have the resolution needed to distinguish the DNA bands in a 2% agarose gel. The individuals used in the association studies (see below) were genotyped, as well, for this polymorphism.

*Fst* size variability typing was performed in the individuals mentioned above, using the PCR amplification conditions described above and specific primers covering the entire *Fst* coding region (FrostF and FrostR) and primers flanking only the PEST region (Frost_PEST_F and Frost_PEST_R, see Table S1) with an annealing temperature of +55°C. Amplification products were run in a 2% agarose gel stained with ethidium bromide using either TAE or SGTB (Grisp, Portugal) buffers.

### PEST domain prediction

PEST regions were predicted using the reference amino acid sequences and the PEST-SCORE algorithm [33]; http://www.at.embnet.org/embnet/tools/bio/PESTfind/.

### Association studies and phenotyping

Two strains (H5 from Lake Hurricane, Mississippi, and W11 from Lake Wappapelo, Missouri) were used to perform an F2 association study. These strains were selected, since, according to the markers used, they show the same polymorphic chromosomal rearrangements, namely, the *X*/*4* fusion, the *Xc,* and *5a* inversions and show differences regarding several traits including chill-coma recovery times. The *X/4* fusion and *Xc* chromosomal rearrangements were typed as described in [34], [35] and [32]. The *4ab* inversion was typed as described in [36] and the *5b* inversion was typed using primers 5bGJ17741F (CCAGCGATAAAGAGAAGA) plus 5bGJ17741R (GCAGGCGGAGATTAGGAC) and the restriction enzyme *Bss*HII (Reis et *al.*, unpublished results). It should be noted that inversions *5a* and *5b* are mutually exclusive. A total of 975 F2 males obtained from three crosses between H5 males and W11 females (F0) were phenotyped for developmental time, chill-coma recovery time, abdominal size and lifespan, but for this work only chill-coma recovery time and abdominal size (as an approximation of total body size) are relevant. The first trait to be measured was developmental time. For this purpose, each of the 83 second generation crosses (F1) were transferred to new flasks every day in order to obtain the precise period of time between oviposition and adult emergence. The resulting F2 males were then individually collected. When F2 males were 10 days old (young adult flies), individual chill-coma recovery times were measured at +25°C after four hours of cold exposure at 0°C. Flies must be able to stand up on their legs in order to be considered completely recovered. Individual photographs were taken when individuals were 20 days old, using a stereomicroscope Nikon ZMS 1500 ®. The resulting JPG files were saved with a resolution of 1600×1200 pixels. Relative abdominal size was estimated by counting the number of pixels in the picture that correspond to this structure, using Adobe Photoshop ®. The flies were then transferred to new vials and kept until they died, in order to measure lifespan. Only males were used in order to avoid potential confounding effects caused by differences between sexes for the traits being studied (see for instance [37]). A total of 453 F2 males corresponding to approximately 66% of the individuals showing the most extreme values for the four phenotypic traits measured were selected to conduct the analyses. Genomic DNA was then extracted and the individuals were genotyped for *Fst* allele sizes (using the primers flanking the PEST region) and the −58 polymorphic site at the 5′ region of the *regucalcin* gene on Muller's element A, as described above. Not all individuals could be genotyped due to unknown reasons. For *Fst* and *regucalcin,* 440 and 422 individuals could be genotyped, respectively.

In the F2 association study there is only one generation of recombination. Therefore associations are expected between the phenotypic traits under study and many polymorphic sites in the region(s) where the causative variant(s) are located. To overcome this issue, 64 individuals (one individual per strain) were phenotyped for chill-coma recovery time and abdominal size, and genotyped for the polymorphic site at the 5′ region of the *regucalcin* gene, as well as for *Fst* allele sizes. Abdominal size could not be determined for four individuals. In this sample, associations between the phenotypic traits under study and polymorphic sites should be found only in the close vicinity of the causative polymorphism.

### Statistical analyses

Genotype – phenotype associations were tested using non-parametric tests and the software SPSS Statistics 17.0 (SPSS Inc., Chicago, Illinois). Using the same software, non-linear as well as linear regression analyses (including a constant) were performed, in order to estimate the amount of phenotypic variation explained by variation in candidate genes.

### Gene expression analyses

*Fst* and *regucalcin* expression levels were determined for three sets of three individuals from the H5 strain, three sets of three individuals from the W11 strain, and three sets of three *D. melanogaster* (Oregon-R) individuals, that were subjected to different conditions, (without chill coma (control), immediately after chill coma recovery (469,8±128,4 seconds for *D. americana* and 1153,3±119,6 seconds for *D. melanogaster*), and 2 hours of recovery after chill coma). Chill coma experiments were performed as described above. It should be noted that all flies are of the same age (10 days old; young adult flies). Immediately after the treatment individuals were frozen in liquid nitrogen. Total RNA was isolated from each set of individuals using TRIzol Reagent (Invitrogen, Spain) according to the manufacturer's instructions and treated with *DNase I* (*RNase*-Free) (Ambion, Portugal). cDNA was synthesized by reverse transcription with SuperScript III First-Strand Synthesis SuperMix for qRT-PCR (Invitrogen, Spain) using random primers. No-template controls and reactions with RNA that was not reverse transcribed were performed in order to confirm the absence of genomic DNA contamination. Highly efficient specific primers (Table S1) for *regucalcin* and *Fst* were used when performing qRT-PCR experiments using the isolated cDNA. Every experiment was performed in duplicate. qRT-PCR was performed with the iQ SYBR Green Supermix (Bio-Rad, Portugal) according to the manufacturer's instructions on a Bio-Rad iCycler with the following program: 3 min at 95°C; 40 cycles of 30 s at 94°C, 30 s at 58°C and 30 s at 72°C followed by a standard melt curve for the *D. americana* strains and 3 min at 95°C; 40 cycles of 30 s at 94°C, 30 s at 60°C and 30 s at 72°C followed by a standard melt curve for the *D. melanogaster* strain. Specific primers (Table S1) were also developed for the endogenous *ribosomal protein L32* (*RpL32*) which was used as the reference gene. Fold change in expression was calculated using the 2^−ΔΔCT^ method [38].

### Likelihood tests of selection

The random-sites models implemented by the PAML 3.15 codeml software [39] have been used. The likelihoods estimated using neutral and positive selection models were compared using a Likelihood Ratio Test: M0 vs. M3, M1a vs. M2a, and M7 vs. M8. The *regucalcin* sequences from the 12 *Drosophila* genomes (http://flybase.org/) and one sequence from *D. americana* (strain NN97.4) were used. The *D. willistoni* sequence was excluded since the change in codon bias reported in this species [40], may affect the inferred phylogeny. For *Fst*, only those species belonging to the *Sophophora* subgenus were used (*D. simulans*, *D. sechellia*, *D. melanogaster*, *D. yakuba*, *D. erecta*, *D. ananassae*, *D. persimilis*, *D. pseudoobscura*), since a reliable alignment could not be obtained when using all species.

For both *regucalcin* and *Fst*, the phylogenetic trees used as input in the codeml analysis were estimated with Maximum Likelihood (ML), using PAUP* v4.0b10 [41]. The evolutionary model used in the ML phylogenetic reconstruction is that suggested by Modeltest 3.7 (Akaike Information Criterion [42]). Trees topology (data not shown) were in general agreement with the view of the group systematics [30]. Heuristic searches were run with the starting tree obtained via stepwise addition and random addition of sequences with 100 replicates. Tree-bisection-reconnection was used as the branch-swapping algorithm. All characters were considered unordered and to have equal weight.

## Results

### The regucalcin gene

In the literature, there is no evidence for the involvement of the *regucalcin* gene (on Mulleŕs element A) in cold tolerance. Indeed, in *D. melanogaster*, Morgan and Mackay [19] and Norry *et al.* [5] did not find any QTL peak for chill-coma recovery on Muller's element A. It is, however, conceivable that the parental lines used in these studies did not segregate *X*-linked alleles for this trait. Therefore, despite these observations, given the high similarity between the regucalcin and the smp-30 proteins (71.9% amino acid identities) we performed F2 association analyses in *D. americana* using the *regucalcin* gene.

In *Drosophila*, temperature resistance is known to be dependent on body size ([43] and references therein), and, as expected, in the F2 association study here performed, using 453 individuals, chill-coma recovery times are negatively linearly correlated with abdominal size (Pearson correlation = −0.290; P<0.0001; Non-parametric Spearman's correlation = −0.263; P<0.0001). The negative correlation between chill-coma recovery times and abdominal size holds when using all the F2 phenotypic data available (N = 974 individuals), although the amount of chill-coma recovery time variability explained by abdominal size is smaller than when using the extremes of the distribution (Pearson correlation  = −0.211; P<0.0001; Non-parametric Spearman's correlation  = −0.160; P<0.0001). Therefore we looked for associations between the C and T variants at the −58 *regucalcin* site polymorphism and both chill-coma recovery times and abdominal size. There are significant associations between this polymorphism and abdominal size (Non-parametric Mann-Whitney Test; P<0.0001) but not with chill-coma recovery time (Non-parametric Mann-Whitney Test; P>0.05). For abdominal size, on average, male flies having the C variant are 5.2% larger than male flies having the T variant (from 0.96 (N = 245) relative units for the T variant to 1.01 (N = 177) relative units for the C variant). Overall, the polymorphism at the *regucalcin* gene explains 3.5% of the variation in abdominal size.

The *regucalcin* polymorphism in the 5′ flanking region (at position −58 relative to the start codon), may be ecologically relevant. Indeed, when using wild-caught individuals, a clear linear correlation between latitude and the frequency of the *regucalcin* T variant at site −58 is observed (Pearson's r = 0.996; P<0.001; Table 1). In *D. americana*, there is a north-south cline for an *X*/*4* fusion – *Xc* inversion chromosomal arrangement that is frequent in the north of the geographic distribution and is almost absent in the south of the distribution [32], [34], [35], [36], [44], and thus it was conceivable that the two gradients were not independent. When the same wild-caught flies are typed for the presence of the *X*/*4* fusion, using the *fused1 Cla*I marker [34], no association is, however, observed between this marker and the presence of the *regucalcin* T variant at position -58 (Fisher's exact test; P<0.05). It should be noted that 79 *X*/*4* fusion and 33 non-fusion chromosomes are being analyzed. Therefore, the gradients for the *X*/*4* fusion and that for the *regucalcin* T variant at position −58 are independent.

**Table 1:** Population frequency of the common *regucalcin* T variant at site −58.

Population	Latitude	Longitude	Frequency	N*
Fremont	41° 26′ N	96° 33′ W	0.824	17
Howell Island	38° 39′ N	90° 42′ W	0.714	28
Lake Wappapelo	37° 8′ N	90° 28′ W	0.594	32
Lake Ashbaugh	36° 15′ N	90° 45′ W	0.538	13
Lake Hurricane	35° 15′ N	91° 40′ W	0.500	4
Saint Francisville	30° 47′ N	91° 29′ W	0.231	13

*sample size.

**Figure 1:**
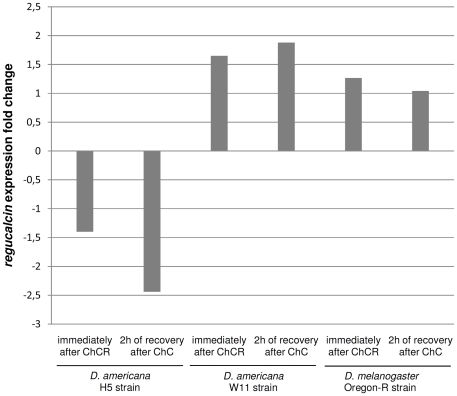
Fold change in *regucalcin* expression after chill-coma recovery (ChCR) obtained by qRT-PCR using the 2^−ΔΔCt^ method. The housekeeping gene *RpL32* was used to normalize the expression values. Expression fold changes were addressed immediately after chill coma recovery and after 2 hours of recovery both for *D. americana* and *D. melanogaster* strains.

The H5 strain (used in the F2 association experiment) is fixed for the T variant and the W11 strain (also used in the F2 association experiment) is fixed for the C variant. Nevertheless for both *D. americana* and *D. melanogaster* no obvious changes in expression levels are detected after cold shock (Fig. 1).

We also looked for signs of positive selection at the *regucalcin* gene at the *Drosophila* genus level. Signs of positive selection are expected at *regucalcin* if amino acid changes at a few sites in the coding region result in changes in cold resistance. The following species were included: *D. melanogaster*, *D. simulans*, *D. sechellia*, *D. yakuba*, *D. erecta*, *D. ananassae*, *D. pseudoobscura*, *D. mojavensis*, *D. virilis, D. americana* and *D. grimshawi*. The analyses revealed no evidence for positively selected amino acid sites (twice the difference of the ln likelihood values is zero and 0.115 (non-significant) when comparing models M1a and M2a and models M7 and M8, respectively; [39]). This result is not unexpected since, in *D. americana* all replacement variants are singletons (based on 18 sequences covering 302 out of the 303 regucalcin amino acids; data not shown).

Chill coma recovery times, relative abdominal sizes, and the *regucalcin* variant at position −58 were determined for 64 unrelated individuals (one individual per strain). In this sample of unrelated individuals a low level of linkage disequilibrium is expected. Therefore, when using such an approach, associations are only expected when the marker used is very close to the causative polymorphism. No association is observed between the *regucalcin* variant at position -58 and chill-coma recovery times or abdominal size (for both Non-parametric Mann-Whitney Test; P>0.05). It should be noted that, in this sample, chill-coma recovery time and abdominal size are as strongly correlated (Pearson correlation = −0.267; P<0.05; Non-parametric Spearman's correlation  = −0.300; P<0.05), as in the F2 association experiment (Pearson correlation = −0.290; Non-parametric Spearman's correlation = −0.263; see above).

### The *Fst* candidate gene

**Figure 2:**
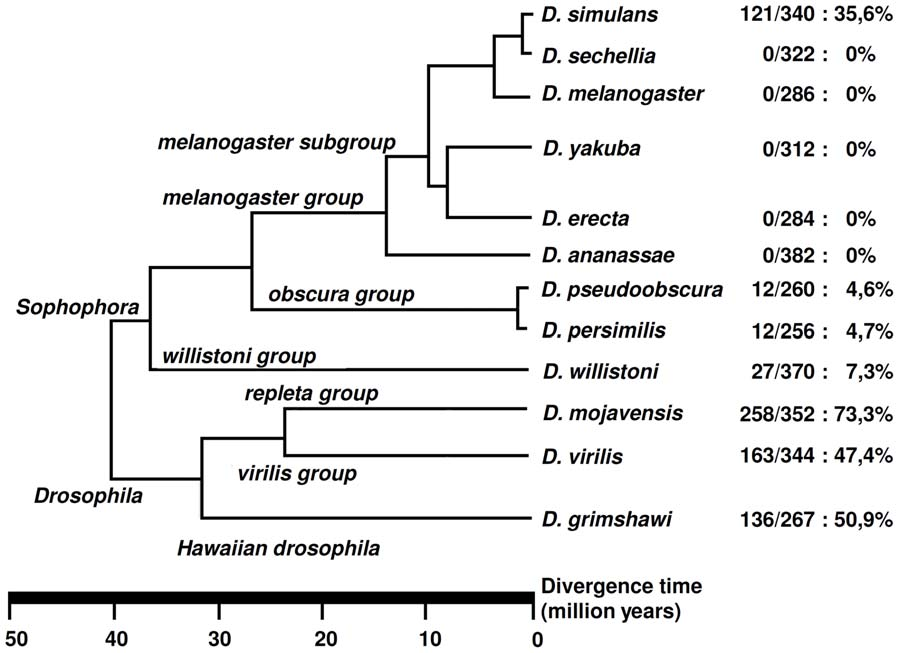
Fst PEST region size differences in *Drosophila*. Numbers after species names indicate, respectively, the total size (number of amino acids) of PEST regions, the size of the Fst protein, and the fraction of the protein containing PEST regions.

The *Fst* gene is located on Muller's element E, and has been shown to be up-regulated in *D. melanogaster*, after chill-coma recovery [10]. In this species, Goto [10] used the PEST-SCORE algorithm [33] to predict the presence of weak PEST regions (putatively involved in rapid protein degradation) in the N-terminal region of the protein. Therefore, we looked for the presence of PEST domains in the Fst protein from 12 *Drosophila* species. Only highly supported PEST region predictions were used, since the biological meaning of weakly predicted PEST regions is unclear [33]. Surprisingly, highly supported PEST regions were detected in seven species but at the C-terminal region of the protein (Fig. 2). Nevertheless, the number and percentage of the amino acid residues that contribute to PEST regions varies widely even in closely related species (Fig 2). For instance, the *D. sechellia* and *D. simulans* Fst sequences differ at 18 amino acid positions. No PEST regions are found in *D. sechellia* because PEST domains must be flanked by a lysine, arginine or histidine [33] and the lysine residues flanking the PEST regions in *D. simulans* are mutated to non-positively charged residues in *D. sechellia*. PEST regions seem to be present in only four species of the *Sophophora* subgenus. In three out of these four species, PEST domains represent less than 10% of the Fst protein sequence. This is in contrast with species from the *Drosophila* subgenus (*D. mojavensis*, *D. virilis* and *D. grimshawi*), where more than 47% of the Fst protein are PEST regions. It is unlikely that such marked differences can be attributed to the use of a single individual per species.

In *D. americana*, seven *Fst* alleles of different sizes were found when using primers FrostF and FrostR and one individual from 64 strains (data not shown). In order to determine the nature of such allele size differences, representatives of each allele size were sequenced (Table 2). Sequencing data indicated that allele size differences are due to a variable number of PEST regions. In this species, PEST regions represent in between 43.3 and 51.3% of the Fst protein depending on the allele size considered.

**Table 2:** *D. americana Frost* size variation.

Individual	Allele size	PEST regions (amino acids) / total number of amino acids
W11	M1	144/321 (44.9%)
NN97.4	M1	151/321 (47.0%)
W4	M1	151/321 (47.0%)
W29	M2	163/348 (46.8%)
NN97.9	M2	170/348 (48.9%)
ML97.3	M2	170/348 (48.9%)
ML97.5	M2	170/348 (48.9%)
NN97.8	M3	186/375 (49.6%)
NN97.2	M3	186/375 (49.6%)
LA18	M3	186/375 (49.6%)
H5	M3	186/375 (49.6%)
HI25	M3	186/375 (49.6%)
H5	M3.5	168/388 (43.3%)
W11	M4	195/402 (48.5%)
LP97.7	M4	202/402 (50.2%)
HI1	M4	202/402 (50.2%)
ML97.4.2	M4	202/402 (50.2%)
HI23	M5	202/422 (47.9%)
LA37	M5	202/422 (47.9%)
W37	M6	234/456 (51.3%)

In the F2 association study here performed, the PEST size genotypes found were homozygous 1 (N = 62), 3 (N = 1), and 4 (N = 18), and heterozygous for allele sizes 1 and 3 (N = 72), 1 and 3.5 (N = 88), 1 and 4 (N = 29), 3 and 3.5. (N = 63), 3 and 4 (N = 63) and 3.5 and 4 (N = 44). This observation reveals that the *D. americana* strains used in the crosses are polymorphic. A significant association between PEST size genotype and chill-coma recovery times was found (Non-parametric Kruskal-Wallis Test; P<0.05), as well as with abdominal size (Non-parametric Kruskal-Wallis Test; P<0.001).

The genotype homozygous for allele size 1 is associated with the slowest chill-coma recovery times (Table 3). On average, male flies homozygous for allele size 1 take 11.6% longer to recover from chill-coma than males from all other genotypic classes (from 429.2 (N = 378) to 478.9 (N = 62) seconds; the two classes explain 1.9% (P<0.005) of the variability observed in the F2 association experiment regarding chill-coma recovery time). On the other hand, *Fst* allele size 3 seems to be associated with large abdominal size (the average for the two categories, namely, not having or having allele size 3, are 0.94 and 1.05 relative units, respectively; Non-parametric Mann-Whitney Test; P<0.001; 17.5% of the variation in abdominal size is explained; P<0.001).

**Table 3:** Average chill-coma recovery time (CCRT), and abdominal size (AS) for *D. americana* male flies with different *Frost* size genotypes.

Genotype	N*	CCRT#	AS#
3/3.5	63	406.5 (376.4–436.6)	1.05 (1.02–1.08)
1/3.5	88	423.9 (402.7–445.0)	0.96 (0.93–0.98)
3/4	63	427.9 (393.7–462.0)	1.05 (1.01–1.09)
1/4	29	428.1 (376.2–480.0)	0.91 (0.87–0.95)
1/3	72	431.1 (405.0–457.1)	1.04 (1.00–1.07)
3.5/4	44	453.0 (417.6–488.5)	0.94 (0.91–0.98)
4	18	468.9 (402.9–534.9)	0.94 (0.90–0.98)
1	62	478.9 (440.5–517.2)	0.91 (0.89–0.93)
3	1	545	1.18

*sample size.

#95% lower and upper boundaries for the mean are given within brackets.

In natural populations, *Fst* allele sizes 3 and 4 are the most common (Table 4). Allele size 4 shows a significant non-linear north-south gradient (Pearson's r = 0.711; P>0.05; non-parametric Spearman's r = 0.886; P<0.05). This could be an indication for a north-south cline for chill-coma recovery or size, since the latter is significantly correlated with chill-coma recovery.

**Table 4:** *Frost* allele size frequencies.

Population*	Fremont	Howell Island	Lake Wappapelo	Lake Ashbaugh	Lake Hurricane	Saint Francisville
Latitude	41° 26′ N	38° 39′ N	37° 8′ N	36° 15′ N	36° 15′ N	30° 47′ N
Longitude	96° 33′ W	90° 42′ W	90° 28′ W	90° 45′ W	91° 40′ W	91° 29′ W
Allele size 0	0	0	0	0	0.056	0
Allele size 1	0	0.056	0.031	0.056	0.056	0
Allele size 2	0.056	0.056	0.109	0.194	0	0.154
Allele size 3	0.527	0.352	0.454	0.444	0.555	0.539
Allele size 3.5	0.028	0.019	0.031	0	0.111	0
Allele size 4	0.333	0.406	0.297	0.250	0.222	0.231
Allele size 5	0.056	0.074	0.078	0.028	0	0.076
Allele size 6	0	0.037	0	0.028	0	0
Sample size	36	54	64	36	18	26

*Populations are shown from north to south.

Chill coma recovery times, abdominal sizes and *Fst* size variation were determined for 64 individuals (one individual per strain). As described earlier, associations between polymorphic sites should have been destroyed by free recombination and therefore associations are only expected to be observed when the marker used is very close to the causative polymorphism. Since many *Fst* allele sizes are present in the sample but most are rare, we analyzed the data by defining two groups of individuals, those that have *Fst* allele size 3 (N = 39) and those that do not have (N = 25). A significant association is observed with abdominal size (Non-parametric Mann-Whitney Test; P<0.05), but not with chill-coma recovery time (Non-parametric Mann-Whitney Test; P>0.05). In this sample, as noted previously, the two variables are as strongly correlated (Pearson correlation  = −0.267; P<0.05; Non-parametric Spearman's correlation = −0.300; P<0.05), as in the F2 association experiment (Pearson correlation  = −0.290; Non-parametric Spearman's correlation = −0.263; see above). This result strongly suggests that the association observed in the F2 association experiment between *Fst* allele sizes and chill-coma recovery is due to body size differences. Surprisingly, given the results obtained in the F2 association study using strains H5 and W11 where *Fst* allele size 3 is associated with large abdominal size, on average, in the sample of 60 unrelated individuals, *Fst* allele size 3 is associated with a 10.1% smaller abdominal size. Therefore, it is not variation in *Fst* allele sizes that contributes to size differences but rather a closely linked polymorphism. Under the assumption that the marker *Fst* allele size 3 is dominant over the other allele sizes, 8.0% of the variation in abdominal size is explained (P<0.05).

In order to address whether other amino acid positions along the Fst protein could be associated with cold resistance, we looked for signs of positive selection at the *Fst* coding region. Signs of positive selection are expected if, amino acid changes at a few Fst amino acid sites result in changes in cold resistance. Analyses for positive selection using random-sites models were run for the *Fst* gene, using the sequences from the sequenced genomes of *D. simulans*, *D. sechellia*, *D. melanogaster*, *D. yakuba*, *D. erecta*, *D. ananassae*, *D. persimilis* and *D. pseudoobscura*. Given the heterogeneity observed for PEST regions from the different species, the alignment used in the present analysis did not include the sequences of the most divergent species *D. virilis*, *D. mojavensis* and *D. grimshawi*, as they were not aligned with confidence. No evidence for sites under positive selection was found in any of the model comparisons (twice the difference of the ln likelihood values is zero when comparing models M1a and M2a, and 0.002118 (non-significant) when comparing models M7 and M8; [39]).

**Figure 3:**
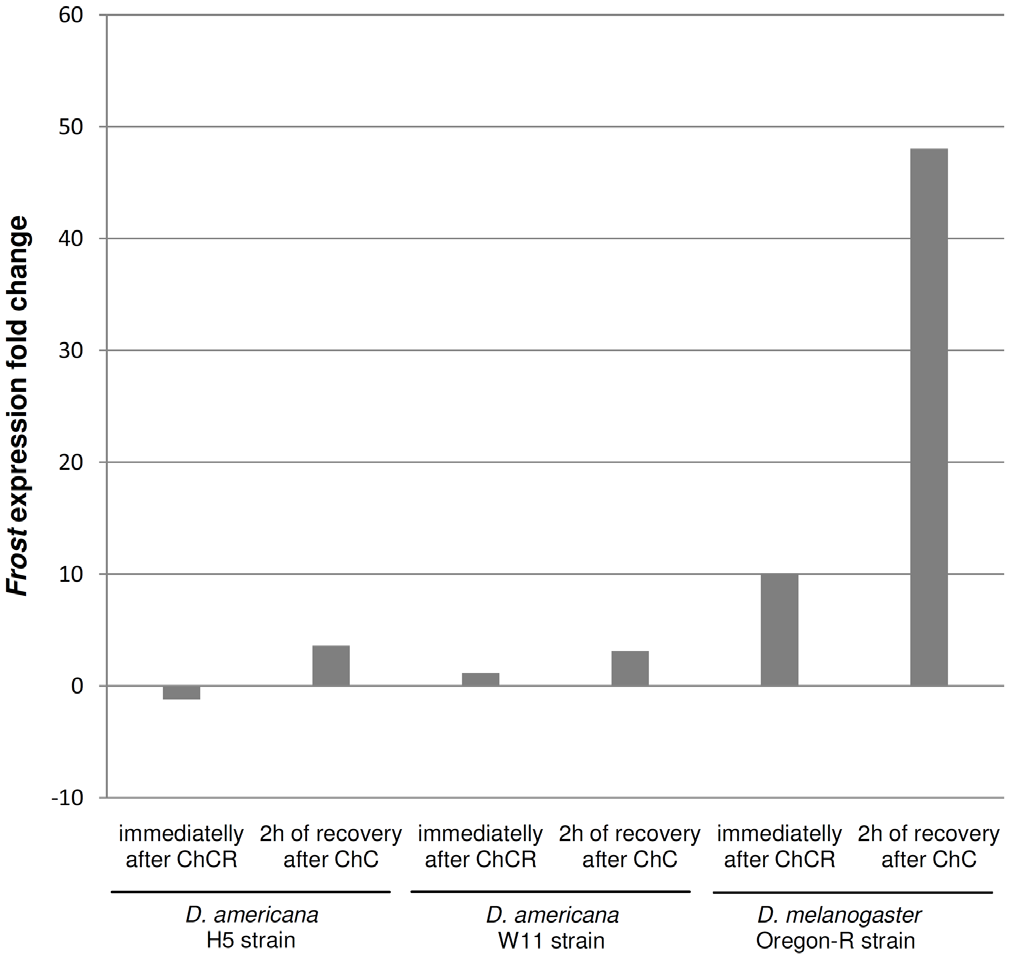
Fold change in *Fst* expression after chill-coma recovery (ChCR) obtained by qRT-PCR using the 2^−ΔΔCt^ method. The housekeeping gene *RpL32* was used to normalize the expression values. Expression fold changes were addressed immediately after chill coma recovery and after 2 hours of recovery both for *D. americana* and *D. melanogaster* strains.

In *D. americana* there are no obvious differences in *Fst* expression levels immediately after chill-coma recovery (Fig. 3). However, after 2 hours of recovery an increase of about 3-fold in *Fst* mRNA levels is observed in both *D. americana* strains H5 and W11. In *D. melanogaster* a 10-fold increase in *Fst* expression was observed immediately after chill coma recovery whereas after 2 hours of recovery a 48-fold increase was observed. Therefore there are important differences in *Fst* expression levels after cold shock in *D. americana* and *D. melanogaster*.

## Discussion

In *D. melanogaster*, the autosomal *Dca* (*smp-30*) gene is up-regulated by cold-shock [12]. Although Rako *et al*. [3] and Telonis-Scott *et al.* [45] failed to find an association between variability at this gene and chill-coma recovery times, Clowers et al. were able to find non-coding polymorphisms that are significantly associated with cold tolerance [11], and thus, this is still one of the most credible candidate genes for cold resistance. Nevertheless, there is no *Dca* gene in species of the *Drosophila* subgenus [13], and thus the *Dca* gene is not a candidate for cold resistance at the whole *Drosophila* genus level. It should be noted that the *Dca* paralogous gene here studied, the *regucalcin* gene on Muller's element A, encodes for a protein that shares 71.9% amino acid identities with Dca, but has never been implicated in cold resistance in *Drosophila*.

In the *D. americana* F2 association study, for the *X*-linked gene *regucalcin*, no association is observed between a common polymorphism in the 5′ flanking region (at position −58 relative to the *regucalcin* start codon) and chill-coma recovery time. Nevertheless, the T variant at position -58 is present at highest frequency in the populations experiencing the lowest average temperatures, and thus those where cold resistance must be most important. Indeed there is a north-south gradient for this polymorphism that is independent of the previously reported north-south *X*/*4* fusion – *Xc* inversion gradient [32], [34], [35], [36], [44]. It should be noted that, for *regucalcin*, we find no evidence for positively selected amino acid sites at the *Drosophila* genus level, and all *D. americana* amino acid variants are singletons. Therefore, changes in expression levels are likely the only way *regucalcin* could be associated with phenotypic differences. Despite this observation, for both *D. americana* and *D. melanogaster*, we find no obvious expression level differences after cold shock. It is conceivable, however, that cold shock does not capture every aspect of cold resistance and thus this gene could still be involved in such a response. In the same F2 association study, an association was, however, observed between the −58 *regucalcin* polymorphism and abdominal size. In this experiment, the T variant that is present at highest frequency in the north of the distribution is associated with small abdominal size. This is in contrast with the commonly reported observation that body size is larger in coldest places (see for instance [46]). Nevertheless, an association could not, however, be detected between this variant and abdominal size when using a sample of unrelated individuals, suggesting that it is variation at another gene located in the same region that is responsible for the observed variation.

Little is known about the function of the *Drosophila* regucalcin protein, and thus we can only speculate about the meaning of the T/C frequency cline. Recently, Kankare *et al.* [47] reported an about 4 fold up-regulation of the *Dca* gene when comparing non-diapausing and diapausing *D. montana* (a member of the *virilis* group of *Drosophila*) females. There is, however, as noted above, no *Dca* gene in species of the subgenus *Drosophila* and thus in *D. montana* [13]. Therefore they likely studied changes in *regucalcin* expression. If so, the *D. americana* north-south gradient for *regucalcin* site −58 could be linked to the propensity to enter diapause. It would be interesting to address whether significant differences in expression level changes are observed in *D. americana* when C/C, T/C and T/T females are compared but this is beyond the scope of this article.

The *Fst* gene shows remarkable variation regarding the size of PEST regions (putatively involved in rapid protein degradation) located in the C-terminal region of the protein encoded by this gene, both within and between *Drosophila* species. The biological meaning of such differences is at present unknown, but this observation suggests that there are important differences in the biological role of *Fst* in different species.

In *D. americana*, we find evidence for associations between PEST region sizes and chill-coma recovery times. It is unknown how differences in PEST region sizes could translate into chill-coma recovery time differences. Nevertheless, associations are also observed between PEST region sizes and abdominal size. Therefore, the association with chill-coma recovery time could be through abdominal size, since, in *D. americana*, the two variables are negatively correlated. When unrelated individuals are used, an association is observed between PEST region sizes and abdominal size, but not with chill-coma recovery times. This result strongly suggests that the association between *Fst* PEST region sizes and chill-coma recovery time is through size. Further studies are needed in order to determine which polymorphism, in the close vicinity of the *Fst* PEST region, could be responsible for the abdominal size differences. The causative polymorphism could be located outside the *Fst* gene. It should be noted that, *Fst* is located inside an intron of the *diuretic hormone* (*Dh*) gene that controls body fluid secretions [48]. It should be noted that, in *D. melanogaster*, Rako *et al*. [3] report that length variation in the *Fst* promoter explains at most 1% of the variation in cold-resistance.

In *D. melanogaster Fst* gene expression is up-regulated during recovery from cold shock [10], [16], [18]. In this species we observe an increase in *Fst* expression levels both immediately after (10-fold) and 2 hours after cold exposure (48-fold), which correlate well with those obtained by Colinet *et al*. [16]. Nevertheless, in *D. americana* we find no obvious expression level differences immediately after cold shock, and we find a 3 fold increase in *Fst* expression levels 2 hours after cold shock. Taken together with the differences regarding the presence of PEST domains, these results suggest that *Fst* gene may have remarkable different roles regarding cold resistance in distantly related *Drosophila* species. In addition to cold tolerance, *Fst* has been reported to respond weakly to abiotic stressors [18], [22], [23], [24], [25] and to be involved in immune response against virus, bacteria and fungi [26], [27], [28], [29]. Therefore, it is conceivable that the relative importance of the different selection pressures vary in the different species.

In conclusion, our work shows that there are significant differences regarding the molecular basis of cold resistance in distantly related *Drosophila* species.

## Supporting Information

Table S1 ***Frost*** **and** ***regucalcin*** **primers used for PCR amplification and sequencing.**
(PDF)
