# Supplementary material for: A Comparative Study of the Short Term Cold Resistance Response in Distantly Related Drosophila Species: The Role of regucalcin and Frost
Source: PLoS One. 2011 Oct 3;6(10):e25520. doi: 10.1371/journal.pone.0025520 (PMC3184994; doi:10.1371/journal.pone.0025520)
Supplement: Table S1 — Frost and regucalcin primers used for PCR amplification and sequencing. (PDF) [file pone.0025520.s001.pdf]

Supplementary Table 1. *Frost* and *regucalcin* primers used for PCR amplification and sequencing

Species	Region analysed	Primers	
Drosophila americana	*Frost*	FrostvirF	CAATGACCMCYTGCTTTC
		FrostvirR	TCTATCGGAACTGACRAT
		Frost_PEST_F	CAGAAGAGCCCGTAGAAA
		Frost_PEST_R	GGTGCCTTGGTAGTCTCG
	frost cDNA	Frost_cDNA_F	CGTAATGGGGATAGATGG
		Frost_cDNA_R	CGTAATGGGGATAGATGG
	regucalcin	DcaF	ATTTTAGTTGGATTCTCG
		DcaR	TTGTGCGTGTGCTTAGAT
	regucalcin cDNA	Dca_amer_ex1	TGATACCGAGGGCAACAT
		Dca_amer_ex2	CGAAGGCAACCGAAGTGA
	RpL32 cDNA	RpL32_RT_F	ACAACAGAGTGCGTCGTC
		RpL32_RT_R	ATCTCCTTGCGTTTCTTC
Drosophila melanogaster	*frost* cDNA	Dmel_Fst_F	TCAGGGTCAGTGGGATGG
		Dmel_Fst_R	CCGTTGGTGGTGGTGGAG
	regucalcin cDNA	Dmel_Regucalcin_F	CCTGCTCCGCTACGACTA
		Dmel_Regucalcin_R	CGAGACGCCATCCCAGTT
	RpL32 cDNA	Dmel_RpL32_F	CGTTTACTGCGGCGAGAT
		Dmel_RpL32_R	CGTTGGGGTTGGTGAGGC
